# The impact of gender and age on bullying role, self-harm and suicide: Evidence from a cohort study of Australian children

**DOI:** 10.1371/journal.pone.0278446

**Published:** 2023-01-05

**Authors:** Kabir Ahmad, Amanda Beatson, Marilyn Campbell, Rubayyat Hashmi, Byron W. Keating, Rory Mulcahy, Aimee Riedel, Shasha Wang

**Affiliations:** 1 School of Business, University of Southern Queensland, Toowoomba, Queensland, Australia; 2 QUT Business School, Queensland University of Technology, Brisbane, Queensland, Australia; 3 School of Early Childhood & Inclusive Education, Queensland University of Technology, Kelvin Grove, Queensland, Australia; 4 School of Business and Creative Industries, University of the Sunshine Coast, Moreton Bay, Queensland, Australia; Swinburne University of Technology, AUSTRALIA

## Abstract

There has been limited longitudinal investigation to date into the association between bullying, self-harm, and suicidality in Australia and the impact of specific demographic differences on this relationship. This is despite the continued rise in the incidence of bullying, self-harm, and suicide. As such, the current study draws on data from the Longitudinal Survey of Australian children (LSAC) to examine the association between bullying, self-harm, and suicidality and explore the impact of demographic differences across three bullying related behaviors (being bullied, bullying others and being both bullied and bullying others). The evidence indicates that bully-victims exhibit the highest risk of self-harm and suicidality in Australia. When considering demographic differences, it was identified that females and adolescents aged 16-17-years-of-age had the highest risk of self-harm and suicidality. Further, a direct curvilinear relationship between age and the categories of self-harm was identified with an inflection point around 16–17 years. The study supports the need for further investigation into the association between bullying, self-harm, and suicidality longitudinally with a particular focus on other moderators.

## Introduction

Self-harm is a significant public health concern [1, 2], with suicide now recognized as the fourth-largest cause of death among 15–19-year-olds worldwide [3, 4]. These issues are of serious concern in Australia also [5], with recent data from the Australian Institute of Health and Welfare (AIHW) reporting that death by suicide among people aged 18–24 years increased by 52% from 10.8 deaths per 100,000 in 2010 to 16.4 deaths in 2020 [6]. In addition, self-harm and suicide have emerged in recent years as the leading cause of disease burden in Australia among young people aged 15–25 [7], resulting in the Prime Minister of Australia declaring self-harm and suicide prevention among young persons to be a national priority [8].

Prior research highlights that bullying, both victimization and perpetration, has been associated with self-harm and suicide-related behaviors among adolescents, even though not all adolescents who are bullied or bullied others will self-harm or consider suicide [9, 10]. This association between bullying, self-harm, and suicide has been observed in children as young as 11- years- old [11] and is concerning as a recent study of Australian school-aged children found that one in seven (14%) students aged 8–14 years old experienced bullying victimization over the preceding 12 months, while 5% of students self-reported bullying others [12]. Given the prevalence of bullying in Australia, the present study aims to better understand the association between bullying, self-harm, and suicide-related behavior among Australian adolescents.

The current study will draw on general strain theory [13], also known as the theory of deviance, to inform an investigation of these relationships. General strain theory predicts that the removal of positive stimuli or the introduction of negative stimuli can cause an individual to commit deviant acts such as revenge or attack others [13]. Although the initial concern of the general strain theory was exclusively on deviant acts targeting others, researchers have extended the theory into the area of negative acts targeting individual selves such as self-harm and suicide [14]. Bullying (both victimization and perpetration) is one of the main sources of strain among adolescents [15, 16], resulting in increased likelihood of self-harm and suicide [17]. Prior research suggests that bullying impacts self-harm and suicide indirectly by undermining self-esteem [18], mental health [19] and social connectedness [20]. Further, a recent systematic review highlights that the impact of socio-demographic factors such as gender and age, though frequently cited in the literature, is inconclusive and in need of future research and replication [21].

Unfortunately, evidence regarding the impact of gender and age on the association between bullying, self-harm, or suicide in Australia is lacking. The few prior studies investigating the association between bullying, self-harm, and suicide have either been limited to one state, focused on one form of bullying, or have used cross-sectional data [19, 22, 23]. To the best of our knowledge, only one prior cross-sectional study has examined the impact of gender and age [24], and only one study has used a cohort-based sample to explore this association among Australian youth [25]. Although this prior work provides preliminary evidence regarding the interplay among bullying, self-harm, and suicide, there is no clear indication as to how these relationships are impacted by gender and age. As the underlying causes of self-harm and suicidal behavior appear to differ across studies [21], and the role and impact of bullying also appear to vary by context [20], there is value in further investigating this complex health issue within Australia [26].

## Research objectives

The current study will add to this scant literature by (1) examining the association between bullying (both victimization and perpetration), self-harm, and suicidality using data from the Longitudinal Survey of Australian children (LSAC) over four years from 2014 to 2018; and (2) exploring the impact of demographic differences, specifically in the area of age and gender. The LSAC dataset captures information on self-harm and suicide-related behavior using a continuum with five categories (self-harm ideation, self-harm attempt, suicide ideation, suicide plan, and suicide attempt). We investigated the association between these five categories and three bullying roles: (i) being bullied (“victim”); (ii) bullying others (“perpetrator”); and (iii) being both bullied and bullying others (“bully-victim”).

The dataset also includes information on children who did not bully and were not victims, as well as a range of demographic variables that have the potential to mitigate the impact of bullying. It is noteworthy that while individual and context-specific risk factors and school-based protective factors have been studied extensively [27], to the best of our knowledge, no previous studies have specifically explored the influence of demographics on the prevalence of bullying, or its association with self-harm and suicidality. This study will add to our understanding of the moderating influence of gender and age within an Australian setting.

## Materials and methods

### Data source and sample selection

The data for this study were obtained from three waves of the K cohort of the Longitudinal Study of Australian Children (LSAC), surveyed in 2014, 2016, and 2018. LSAC is a nationally representative biennial survey of Australian households that gathers information about the development and welfare of children in areas such as parenting, family, peers, education, childcare, and health [28]. The LSAC study follows a multi-stage, stratified, and clustered sampling design to select participants. The household is the primary sampling unit from where the children and their caregivers are selected. We direct interested readers to Soloff et al. for further details regarding the LSAC survey design and methodology [29].

The K cohort of LSAC was recruited in 2004 while the children were 4–5 years old. The present study analyzed data on children’s bullying victimization, bullying perpetration, and self-harm or suicide-related behavior from 2014 (wave 6) when the K cohort’s participants were 14–15 years old, to 2018 (wave 8) when the respondents were 18–19 years old. The sample consisted of children who responded to questions about bullying, self-harm, and suicide-related behaviors. There were 3,315 children of age 14–15 years (Wave 6), 2913 children of age 16–17 years (Wave 7), and 2,641 children of age 18–19 years (Wave 8) in the study sample. Over the three waves of this panel data, the total observations were 8,869 from 3,604 unique children with an average of 2.5 observations per child.

### Ethics statement

The Australian Institute of Family Studies Ethics Committee reviewed and approved each wave of the LSAC study. The ethics committee is registered with the National Health and Medical Research Council (NHMRC) and has ensured that the LSAC project fulfills the Australian National Statement on Ethical Conduct in Human Research. The data custodians authorized the current study, and as the present study only utilized secondary de-identified data, no additional ethics approval was necessary.

### Measures used in the study

Measures of self-harm and suicide-related behaviors were used as outcome variables in this study. The measures for self-harm among children were derived from the AVON Longitudinal Study of Parents and Children (ALSPAC): Life of a 16+ Teenager questionnaire [30]. Measures for suicide-related behaviors were derived from the National Survey of Mental Health & Wellbeing [31].

#### Self-harm

Self-harm ideation was assessed with the question: “During the last 12 months have you thought about hurting yourself on purpose in any way (i.e., by taking an overdose of pills, or by cutting or burning yourself)?” To assess self-harm attempts, the following question was asked: “During the past 12 months have you hurt yourself on purpose in any way (i.e., by taking an overdose of pills, or by cutting or burning yourself)?” Binary variables (i.e., 1 for yes and 0 for no) were used to capture these responses.

#### Suicide-related behaviors

To assess suicide ideation the following question was asked: “During the past 12 months did you ever consider attempting suicide?” Suicidal planning was assessed with the following question: “During the past 12 months did you make a plan about how you would attempt suicide?” Suicide attempt was assessed with the following question: “During the past 12 months, how many times did you attempt suicide?” All responses were again coded as binary variables.

#### Bullying (exposure variable)

In wave 6, adolescents were asked eleven questions and in wave 7 they were asked nine questions to assess their experience of bullying. These items were drawn from the School Climate Bullying Scale from the Edinburgh Study of Youth Transitions and Crime, with minimal modification for the Australian context [31–34]. In wave 8, the adolescents were asked eight questions to assess their experience of being victimized. To assess bullying victimization, we followed the direction of previous research [2, 22], a dichotomous variable was created to indicate if any children had experienced any form of bullying (i.e., 1 for yes and 0 for no).

Participants were also asked in all three waves whether they had bullied anyone, with this data used to create a dichotomous variable for participants who bullied other children. A third dichotomous variable: “bully-victims” was created for participants who experienced victimization but also bullied others. The final dichotomous variable captured the balance of the participants—those that were neither bullied nor perpetrated bullying. The four dichotomous variables were created to be mutually exclusive (i.e., bully-victims were not reported in the other two categories).

#### Socio-demographic variables

This study identified the following socio-demographic control variables: (i) gender of the children (male or female), and (ii) age of the children (14 to 19 years).

### Potential sources of bias

While cohort-based studies are less prone to bias than other observational approaches (e.g., cross-sectional studies), three sources of potential bias in cohort studies have been highlighted as warranting attention, selection bias, informational bias and confounder bias [35]. Specifically, bias is purported to arise from the selection of cases (i.e., selection bias), the loss of information between waves of data collection (i.e., informational bias), and confusion around the explanation of observed effects, particularly where a causal relationship is confounded (i.e., confounder bias).

Concerning selection bias, there are three key considerations: (i) ensuring that the sample includes exposed and non-exposed individuals; (ii) taking care to control for geographical differences across populations that could influence generalisability; and (iii) making sure that data is not biased by the absence (i.e., non-response) of groups within the population. All three sources of selection bias are controlled for within this study. We included data from both the bullying and non-bullying populations, and the data collection procedures for the LSAC study comply with the highest international standards for the conduct of longitudinal cohort studies, and intentionally seek to eliminate the bias associated with geographic and non-response (see Sanson A, 2003 or Gray & Sanson, 2005 for a more detailed explanation) [36, 37]. We considered the index of relative socio-economic disadvantage (IRSD) for controlling the analytical models. This index is a decile index that summarises economic and social conditions of people and households within an area and includes only measures of relative disadvantage, where a low score indicates relatively greater disadvantage in general [38].

Informational bias was controlled for during the LSAC data collection process, with meticulous care taken to capture relevant data across waves and to track respondents between waves. In this particular study, this is less of an issue as we analyze aggregate-level data across the three waves of the LSAC dataset. While it is difficult to anticipate all possible sources of confounder bias, we sought to reduce the potential for confounder bias by undertaking interaction analysis to examine the influence of a potential confounders (i.e., demographics) on the relationship between bullying, self-harm, and suicide-related behavior.

### Statistical analysis

All analyses were performed using the statistical software Stata (version 17). Descriptive statistics (frequency and percentage) are provided in Table 1 for the characteristics of the study participants across all three waves. Longitudinal random-effects logistic regression was used to examine the association between the types of bullying behavior, self-harm, and suicidal behaviors. This regression approach is appropriate as it allows for the modeling of main effects for variables that change over time (e.g., age of respondents [39]). Table 2 presents the results for the five base models, and Table 3 presents adjusted models across the different categories of the self-harm continuum. These models consider the impact of the students’ role in bullying on self-harm and suicidality, and control for the influence of demographics. The test results are displayed as odds ratios with 95% confidence intervals (CIs) and p-values provided for main effects. A predictor was considered statistically significant only if the respective p-value was less than or equal to 0.05.

**Table 1:** Characteristics of the study participants and the sample.

Variables	Wave 6 (n = 3315)		Wave 7 (n = 2913)		Wave 8 (n = 2641)	
	n	%	n	%	n	%
Sex of child						
*Male*	1694	51.1	1478	50.7	1330	50.4
*Female*	1621	48.9	1435	49.3	1311	49.6
Age of child						
*14-years-old*	1952	58.9				
*15-years-old*	1363	41.1				
*16-years-old*			1610	55.3		
*17-years-old*			1303	44.7		
*18-years-old*					1532	58.0
*19-years-old*					1109	42.0
Mothers’ Education						
*Year 12 or less*	779	23.5	713	24.5	1087	41.2
*Certificate*	1024	30.9	823	28.3	542	20.3
*Graduate Diploma/Diploma*	1209	36.5	1091	37.5	785	29.7
*Postgraduate*	303	9.1	286	9.8	227	8.6
Mothers’ employment						
*Employed*	2671	80.6	2380	81.7	1643	62.2
*Unemployed*	73	2.2	57	1.9	48	1.8
*Not in labour force/others*	571	17.2	476	16.4	950	36.0
SEIFA Index of Relative Socio-economic Disadvantage (mean)	5.98		6.03		6.14	

**Table 2:** Base models predicting self-harm and suicide-related behaviors.

	Model-1: Self-harm ideation	Model-2: Self-harm attempt	Model-3: Suicidal ideation	Model-4: Suicide plan	Model-5: Suicide attempt
Variables	OR (95% CI), p-value	OR (95% CI), p-value	OR (95% CI), p-value	OR (95% CI), p-value	OR (95% CI), p-value
Bullying role					
*Not involved (ref*.*)*					
*Victim only*	**3.69 (2.98–4.57), <0.001**	**3.34 (2.56–4.37), <0.001**	**2.83 (2.19–3.67), <0.001**	**2.99 (2.24–3.99), <0.001**	**2.27 (1.59–3.24), <0.001**
*Perpetrator only*	**2.02 (1.31–3.13), 0.001**	1.72 (0.96–3.08), 0.067	1.35 (0.76–2.38), 0.307	1.72 (0.94–3.16), 0.079	0.68 (0.26–1.8), 0.441
*Bully-victims*	**5.49 (4.44–6.79), <0.001**	**4.74 (3.65–6.16), <0.001**	**4.46 (3.48–5.72), <0.001**	**4.95 (3.76–6.5), <0.001**	**3.51 (2.51–4.92), <0.001**

Notes: Significant interactions are shown in bold.

**Table 3:** Adjusted base models predicting self-harm and suicide-related behaviors.

	Model-1: Self harm ideation	Model-2: Self-harm attempt	Model-3: Suicidal ideation	Model-4: Suicidal plan	Model-5: Suicidal attempt
Variables	OR (95% CI), p-value	OR (95% CI), p-value	OR (95% CI), p-value	OR (95% CI), p-value	OR (95% CI), p-value
**Bullying role**					
Not involved (ref.)					
*Victim only*	**3.55 (2.86–4.39), <0.001**	**3.09 (2.37–4.04), <0.001**	**2.85 (2.19–3.71), <0.001**	**2.99 (2.24–4.01), <0.001**	**2.21 (1.55–3.16), <0.001**
*Perpetrator only*	**2.35 (1.52–3.64), <0.001**	**1.86 (1.04–3.34), 0.037**	1.55 (0.87–2.76), 0.137	**1.92 (1.04–3.55), 0.037**	0.72 (0.27–1.91), 0.510
*Victim-perpetrator*	**6.66 (5.36–8.28), <0.001**	**5.28 (4.05–6.89), <0.001**	**5.39 (4.16–6.99), <0.001**	**5.80 (4.37–7.71), <0.001**	**3.91 (2.77–5.51), <0.001**
Sex of the child					
*Male (ref*.*)*					
*Female*	**4.39 (3.54–5.43), <0.001**	**4.04 (3.14–5.20), <0.001**	**2.07 (1.62–2.64), <0.001**	**1.70 (1.32–2.18), <0.001**	**1.91 (1.39–2.62), <0.001**
Age					
*14-15-years-old (ref*.*)*					
*16-17-years-old*	**1.72 (1.45–2.04), <0.001**	**1.40 (1.14–1.73), 0.001**	**1.84 (1.49–2.27), <0.001**	**1.55 (1.24–1.95), <0.001**	**1.55 (1.16–2.06), 0.003**
*18-19-years-old*	**1.62 (1.35–1.94), <0.001**	1.17 (0.93–1.47), 0.161	**2.00 (1.60–2.51), <0.001**	**1.87 (1.47–2.37), <0.001**	**1.49 (1.09–2.02), 0.011**

Notes: Significant interactions are shown in bold. Models are controlled for social disadvantage index score

In addition to the analysis of the main effects, the findings of separate regression models are presented for interactions between bullying role and demographics across the five components of the self-harm continuum. The findings of this analysis are presented in Appendix B.

## Results

A total of 8,869 person-year observations from 3,604 participants were included in the analysis. Across the waves, around 49% of respondents were female and 60% were from the metro area. Among their parents, over 80% of the mothers of participating children were employed in Wave 6 and 7, while this dropped to around 60% in Wave 8. About 75% of mothers also had tertiary qualifications (from certificate course to post-graduate degree) in Wave 6 and 7, with this dropping to 60% in Wave 8. The differences in employment and education between Waves 7 and 8 were believed to be due to respondent attrition. The pooled prevalence of employed mothers (75.5%) of the sample is very close to the general population, as ABS reported 73.7% employed mothers in couple families with children under 15 in June 2021. The relative disadvantage of economic and social conditions of living areas among children was less and consistent across waves (on average 6 out of 10), as indicated by the IRSD index of the Socio-Economic Indexes for Areas (SEIFA) [38]. The complete characteristics of the study participants are provided in Table 1.

Table 2 reveals a strong connection between bullying roles and the five categories of self-harm and suicide-related behaviors. The odds ratios presented are for the reference category for those that did not experience or perpetrate any bullying (not involved). From this data, we can see that adolescents who were bully-victims were observed to have the highest risk across all five categories of the self-harm continuum, followed by victim-only and perpetrator-only respondents. For example, we can see from Table 2 that bully-victims exhibited the highest risk for self-harm ideation (OR 5.49, 95% CI: 4.44–6.79), more than five times the risk compared to persons that were not involved in bullying. The odds of bully-victims experiencing self-harm ideation were 2.7 times higher than perpetrators (OR 2.02, 95% CI: 1.31–3.13) and 1.5 times higher than victims (OR 3.69, 95% CI: 2.98–4.57) within this category. Similar patterns (i.e., risk ratios between bullying roles) were visible across the other four categories of the self-harm continuum, although the risk was generally smaller as we moved along the continuum toward the more negative outcomes (i.e., suicide attempts). Caution needs to be exercised when interpreting the risks associated with perpetrator-only individuals, however, as the odds ratios were only statistically significant for the self-harm ideation category.

Table 3 presents data related to adjusted versions of the basic regression models presented in Table 2 based on gender and age. These new models provide strong evidence for gender and age differences, with females exhibiting higher odds across all categories of the self-harm continuum. The most profound difference was observed in relation to self-harm behaviors with females around four times more likely than males to experience self-harm ideation (OR 4.39, 95% CI: 3.54–5.43) or attempt self-harm (OR 4.04, 95% CI: 3.14–5.20). Though less in effect size, significantly, females were also observed to be around two times more likely than males to report suicide-related behaviors.

Age was also observed to have an impact on the different categories of the self-harm continuum. Respondents in the 16–17 years age group were observed to be, on average, around 1.5 times more likely to report self-harm or suicide-related behaviors, with the strongest effect observed for reporting suicidal ideation (OR 2.00, 95% CI: 1.60–2.51) followed by suicidal plan (OR 1.87, 95% CI: 1.47–2.37). The findings revealed that adolescents aged 14–15 (reference group) were generally observed to have the lowest associations with the three suicide-related behaviors, while the older respondents aged 18–19 years had the lowest odds for the two self-harm categories.

Building on the findings presented in Table 3, further analysis was undertaken to examine whether the impact of bullying role on the five categories of the self-harm continuum was moderated by demographics. The results for the associated interactions are provided in Appendix B. While similar patterns were observed to those discussed above (i.e., females were more likely to report self-harm and suicide than males, 16–17-year-olds seemed more susceptible to self-harm and suicide generally), there were nevertheless, some interesting exceptions observed within and across the five categories of the self-harm continuum.

For example, while females were identified to have the greatest risk across all categories of self-harm and suicide-related behaviors, the most profound impact was observed for female bully-victims in the self-harm categories. Female respondents reported 24 times the likelihood of a self-harm attempt (OR 24.04, 95% CI: 17.01–33.95) and 18 times the risk of self-harm ideation (OR 18.16, 95% CI: 11.97–27.54) compared to males that were not involved (reference category). Female bully-victims were more than 5 times as likely to have self-harm ideation than male bully-victims (OR 4.53, 95% CI: 3.31–6.21) or to attempt self-harm (OR 3.17, 95% CI: 2.12–4.75). Female bully-victims were also twice as likely to report self-harm ideation (OR 12.31, 95% CI: 8.83–17.17) or attempt self-harm (OR 8.85, 95% CI: 589–13.30) than female victims, and around four times as likely to report self-harm behaviors than female perpetrators. Though female bully-victims were more likely to be observed across all categories of the continuum, the relative risk reduced as we moved up the continuum. This is most visible at the two extremes of the continuum, where female bully-victims (OR 6.02, 95% CI: 3.66–9.90) had around twice the risk of suicide attempts than female victims (OR 3.31, 95% CI: 2.02–5.43) and male bully-victims (OR 2.48,95% CI: 1.54–3.98).

Bully-victims aged 16–17 years were the most likely to report self-harm ideation (OR 12.49, 95% CI: 8.59–18.14) and attempt self-harm (OR 8.65, 95% CI: 5.44–13.76), with these odds more than 1.3 times the risk of self-harm ideation for victims (OR 9.47, 95% CI: 6.29–14.26), but only 8% higher than the risk of self-harm attempts for victims (OR 8.03, 95% CI: 4.84–13.32). Likewise, bully-victims aged 16–17 years were more than twice as likely to report self-harm ideation (OR 4.78, 95% CI: 2.44–9.33) or attempt self-harm (OR 3.50, 95% CI: 1.46–8.34) than perpetrators the same age. Bully-victims aged 18–19 years displayed the greatest absolute risk of suicidal ideation (OR 11.48, 95% CI: 6.8–19.38) and suicide planning (OR 12.09, 95% CI: 6.86–21.32). The suicide attempt category reverted to the patterns observed for the self-harm categories with the greatest absolute and relative risk observed for 16–17-year-old bully-victims.

## Discussion and conclusions

The prevalence of self-harm and suicide-related behaviors among adolescents in Australia is on the increase and reducing its prevalence and impact has emerged as a major health priority. The current study provides valuable guidance to policy-makers as they seek to respond by reporting on the association between bullying role and the five categories of a self-harm continuum (self-harm ideation, self-harm attempt, suicidal thought, suicidal plan, suicide attempt) using data from a large cohort study of Australian children. The findings demonstrate that involvement in bullying as either a victim, perpetrator or both is associated with an elevated risk of self-harm ideation, with victims and bully-victims more likely to attempt self-harm and ideate, plan or attempt suicide. To gain additional insight, we investigated the influence of demographic factors. The findings from this analysis make several interesting contributions to our understanding of how different bullying roles impact self-harm and suicidality among Australian adolescents.

### Examining the association between bullying, self-harm, and suicidality

In relation to the first research objective, the current study demonstrated that among the three investigated bullying roles, bully-victims exhibited the highest risk of self-harm and suicidality. This observation persisted even after controlling for various demographic factors. While this observation is consistent with general strain theory and suggests that any involvement in bullying will result in increased strain [13], it is clear that bully-victims experience the greatest strain, and subsequently, have less favorable outcomes than either bullies or victims. This finding concurs with the findings of a recent multi-level meta-analysis [40] which found that bully-victims had higher odds of suicidal ideation and behavior than either victims or bullies. The historic argument in support of this association is that students who are both bullied and bully others often come from a disadvantaged background [27] and therefore are prone to greater internalization and higher levels of anxiety and depression [41]. From the interpersonal theory lens, victims of bullying normally experience lower belongingness which is a direct cause of their desire for death [42, 43]. However, emerging evidence suggests that a key contributing factor could be the reluctance of this particular group to seek help [44].

Our research adds to the scant literature by presenting evidence of how a students’ role in bullying impacts both suicidal and non-suicidal self-injury. While prior research has reported an association between victimization and self-harm [45], the current study is the first to explore the impact of different bullying roles across an expanded self-harm continuum. Our paper also extends an earlier single-wave study of Australian children using the LSAC dataset [3] by using data from three waves of the LSAC dataset to explore how the association between bullying role, self-harm, and suicidality is influenced by different demographic factors. Specific contributions related to this extension will be the focus of the remaining discussion.

### Exploring the impact of gender and age differences

In relation to the second research objective, the current study provides a more nuanced understanding of how biological sex and age impact self-harm and suicidality among adolescents. While the findings reveal that females and adolescents aged 16-17-years-of-age had the highest risk of self-harm and suicidality, there were some interesting differences across the categories of the continuum. Contrary to the findings of a recent meta-analysis which found no statistical association between bullying roles and biological sex [46], the current study found a strong positive association for females across all three bullying roles, with female bully-victims statistically more likely than male bully-victims to report self-harm and suicide-related behaviors. Though an earlier meta-analysis did find evidence of a weak gender effect for victims and a small effect for bullies and bully-victim [47], this earlier study found that male adolescents were more likely to engage in bullying. This finding adds to the sparse literature examining gender and its relationship to bullying, self-harm and suicide in Australia.

While there is an abundance of literature on the differences been the bullying behaviour of girls and boys [48], there is scant research on any gender nuanced prevention or intervention strategies. As most bullying behaviours are amongst the same gender, this provides a significant opportunity to research whether targeted prevention programs for girls and boys separately would reduce bullying behaviours. As it has been shown that girls who feel comfortable talking to adults about bullying were less likely to be involved in any bullying behaviours [49], then providing girls with more opportunity to talk with adults could be one solution to assist these girls.

The current study also provides evidence for a direct curvilinear relationship between age and the five categories of the self-harm continuum, with an inflection point around 16–17 years. This curvilinear relationship was also present when age interacted with the bullying role, however, it was in stark contrast with those that had no experience with bullying where age was observed to have a positive monotonic relationship with self-harm and suicidality. This finding could help to explain some of the inconsistent results reported in the literature. For example, a meta-analysis [47] revealed a strong association between victimization, perpetration, and self-harm, but other studies have found no association between age and bullying role [50]. It might be possible that a similar curvilinear relationship was either masking or ameliorating some of these effects in the earlier studies. Interestingly, the possibility of a curvilinear relationship between age and bullying was first hinted at in the context of cyberbullying [51], however, the current study is the first to explicitly examine the nature of this relationship in the context of bullying roles, self-harm, and suicidality.

### Limitations and future research directions

Although this current study has provided insight into how the relationship between bullying role, self-harm, and suicidality is influenced by a range of demographic factors; there are nevertheless some limitations that need to be acknowledged. One limitation is the lack of knowledge about whether the respondents were exposed to anti-bullying programs or other additional risk or preventative factors before their involvement in LSAC. The current study also did not distinguish between waves of data collection, or between traditional bullying and cyberbullying, or consider specific types of bullying (e.g., physical, verbal, cyberbullying, etc.). Future studies could investigate whether the findings of the current study differ across waves using trajectory analysis or examine whether there are differences between traditional bullying and cyberbullying, and whether these findings are sensitive to different types of bullying.

Prior research examining bullying among adolescents in Australia using the LSAC dataset also suggests that care needs to be taken when generalizing the findings of this dataset to other international settings, as the Australian population is comparatively wealthy and advantaged even by OECD standards [3]. Future studies could expand the focus and control for sources of geographical bias to enhance the generalizability of the findings.

LSAC is a secondary data set. While it has several merits such as sample size and its longitudinal nature, it also has limitations like other secondary data sets. For example, the authors are not able to change the measurements. The data set only contains binary (yes/no) single-item variables which may reduce the richness of the results. Future researchers could introduce multiple items measures using interval scales in their surveys.

Finally, with suicide being the leading cause of death among adolescents in Australia, it is also important to gain further understanding of potential correlates to self-harm and suicidality, and whether risk and protective factors act to moderate the impact of different bullying roles on self-harm and suicidality. This research would aid policymakers to more effectively intervene to reduce the negative outcomes associated with bullying [52]. Building on the findings of the current study, future research could also explore the impact of risk-reduction programs that try to reduce the prevalence and harm associated with bullying.

Prior research suggests that these anti-bullying programs need improvement [53], and the findings of the current study can provide some clues on where policymakers need to focus, particularly as children grow older as traditional interventions have been shown to wane in effectiveness over time [54]. The results of the current study may assist by reinforcing the importance of targeting such interventions to certain groups (e.g., female bully-victims). Of particular importance is the need to understand how to engage parents and families as part of the solution to support these groups [55].

## Supporting information

S1 Appendix Interactions with bullying roles to predict self-harm and suicide-related behaviors. (DOCX)

S1 Table PRISMA-P 2015 checklist. (DOCX)
