# Supplementary material for: The impact of gender and age on bullying role, self-harm and suicide: Evidence from a cohort study of Australian children
Source: PLoS One. 2023 Jan 5;18(1):e0278446. doi: 10.1371/journal.pone.0278446 (PMC9815580; doi:10.1371/journal.pone.0278446)
Supplement: S1 Appendix — (DOCX) [file pone.0278446.s001.docx]

**Appendix A:** Interactions with bullying roles to predict self-harm and suicide-related behaviors

|  | Model-1: Self-harm ideation | Model-2: Self-harm attempt | Model-3: Suicidal ideation | Model-4: Suicide plan | Model-5: Suicide attempt |
| --- | --- | --- | --- | --- | --- |
| Variables | OR (95% CI), p-value | OR (95% CI), p-value | OR (95% CI), p-value | OR (95% CI), p-value | OR (95% CI), p-value |
| Bullying role # Sex of child |  |  |  |  |  |
| *Not involved # Male (ref.)* |  |  |  |  |  |
| *Not involved # Female* | **3.16 (2.25 - 4.44), <0.001** | **2.55 (1.66 - 3.91), <0.001** | **1.53 (1.02 - 2.29), 0.038** | 1.15 (0.73 - 1.79), 0.545 | 1.17 (0.68 - 1.99), 0.576 |
| *Victim only # Male* | **2.58 (1.78 - 3.72), <0.001** | **2.4 (1.5 - 3.84), <0.001** | **2.12 (1.39 - 3.22), <0.001** | **2.25 (1.43 - 3.54), <0.001** | 1.46 (0.82 - 2.62), 0.202 |
| *Victim only # Female* | **12.31 (8.83 - 17.17), <0.001** | **8.85 (5.89 - 13.3), <0.001** | **4.85 (3.33 - 7.07), <0.001** | **4.02 (2.68 - 6.04), <0.001** | **3.31 (2.02 - 5.43), <0.001** |
| *Perpetrator only # Male* | **2.47 (1.35 - 4.53), 0.003** | 1.63 (0.69 - 3.84), 0.261 | 1.08 (0.48 - 2.41), 0.859 | 1.19 (0.49 - 2.87), 0.702 | 0.36 (0.07 - 1.72), 0.199 |
| *Perpetrator only # Female* | **5.99 (3.14 - 11.41), <0.001** | **5.03 (2.21 - 11.44), <0.001** | **2.81 (1.22 - 6.44), 0.015** | **2.99 (1.27 - 7.05), 0.012** | 1.46 (0.41 - 5.25), 0.561 |
| *Bully-victims # Male* | **4.53 (3.31 - 6.21), <0.001** | **3.17 (2.12 - 4.75), <0.001** | **3.75 (2.63 - 5.35), <0.001** | **3.81 (2.61 - 5.59), <0.001** | **2.48 (1.54 - 3.98), <0.001** |
| *Bully-victims # Female* | **24.04 (17.01 - 33.95), <0.001** | **18.16 (11.97 - 27.54), <0.001** | **8.65 (5.89 - 12.7), <0.001** | **7.77 (5.16 - 11.69), <0.001** | **6.02 (3.66 - 9.9), <0.001** |
| Bullying role # Age |  |  |  |  |  |
| *Not involved # Age 14-15 (ref.)* |  |  |  |  |  |
| *Not involved # Age 16-17* | **2.11 (1.44 - 3.1), <0.001** | **1.73 (1.05 - 2.86), 0.033** | **2.07 (1.25 - 3.42), 0.005** | 1.69 (0.95 - 3), 0.075 | **1.93 (1 .00- 3.73), 0.05** |
| *Not involved # Age 18-19* | **2.38 (1.64 - 3.46), <0.001** | **2.07 (1.28 - 3.35), 0.003** | **3 (1.86 - 4.83), <0.001** | **3 (1.77 - 5.1), <0.001** | **2.35 (1.24 - 4.45), 0.009** |
| *Victim only # Age 14-15* | **5.1 (3.44 - 7.56), <0.001** | **4.85 (2.96 - 7.94), <0.001** | **3.41 (2.04 - 5.72), <0.001** | **4.38 (2.5 - 7.65), <0.001** | **2.73 (1.37 - 5.44), 0.004** |
| *Victim only # Age 16-17* | **9.47 (6.29 - 14.26), <0.001** | **8.03 (4.84 - 13.32), <0.001** | **8.69 (5.2 - 14.54), <0.001** | **6.8 (3.84 - 12.05), <0.001** | **5.5 (2.76 - 10.96), <0.001** |
| *Victim only # Age 18-19* | **7 (4.77 - 10.28), <0.001** | **4.45 (2.73 - 7.26), <0.001** | **6.84 (4.19 - 11.16), <0.001** | **6.52 (3.79 - 11.23), <0.001** | **4.62 (2.39 - 8.93), <0.001** |
| *Perpetrator only # Age 14-15* | **3.42 (1.65 - 7.11), 0.001** | 2.39 (0.86 - 6.64), 0.094 | 1.92 (0.69 - 5.33), 0.209 | 1.69 (0.49 - 5.79), 0.406 | 1.07 (0.2 - 5.87), 0.936 |
| *Perpetrator only # Age 16-17* | **4.78 (2.44 - 9.33), <0.001** | **3.5 (1.46 - 8.34), 0.005** | **4.06 (1.73 - 9.52), 0.001** | **5.48 (2.26 - 13.3), <0.001** | 0.92 (0.17 - 4.82), 0.919 |
| *Perpetrator only # Age 18-19* | 2.37 (0.82 - 6.8), 0.109 | 2 (0.5 - 8.02), 0.330 | 2.03 (0.5 - 8.25), 0.324 | 2.57 (0.62 - 10.69), 0.193 | 2.1 (0.36 - 12.36), 0.413 |
| *Bully-victims # Age 14-15* | **7.9 (5.46 - 11.44), <0.001** | **7.02 (4.43 - 11.14), <0.001** | **7.27 (4.54 - 11.63), <0.001** | **7.51 (4.48 - 12.61), <0.001** | **5.77 (3.1 - 10.71), <0.001** |
| *Bully-victims # Age 16-17* | **12.49 (8.59 - 18.14), <0.001** | **8.65 (5.44 - 13.76), <0.001** | **10.93 (6.83 - 17.49), <0.001** | **11.09 (6.62 - 18.59), <0.001** | **7.59 (4.07 - 14.14), <0.001** |
| *Bully-victims # Age 18-19* | **11.89 (7.81 - 18.12), <0.001** | **7.16 (4.2 - 12.21), <0.001** | **11.48 (6.8 - 19.38), <0.001** | **12.09 (6.86 - 21.32), <0.001** | **5.17 (2.53 - 10.58), <0.001** |

Notes: Results presented relate to separate models for each interaction. Insufficient data indicates that the specific interaction effect was not observable due to low data counts in that category. Significant interactions are shown in bold.
